# Five years review of extraction frequencies at S.D.M. College of dental sciences and Hospital in orthodontic department

**DOI:** 10.4317/jced.56264

**Published:** 2019-11-01

**Authors:** Shanthiprasad Indra, Arun Kumar, Niranjanaprasad Indra, GC Ramesh, Ganesh Chinthan, Sachin Bharadwaj

**Affiliations:** 1 Senior Lecturer, Department of Orthodontics & Dentofacial Orthopedics, Sharavathi Dental College & Hospital, Shivamogga, India; 2 Professor and Head of the Department, Department of Orthodontics & Dentofacial Orthopedics, Sharavathi Dental College & Hospital, Shivamogga, India; 3 Associate Professor, Department of Oral and Maxillofacial Surgery, Institute of Dental Sciences, Bareilly, Uttar Pradesh, India; 4 Professor, Department of Orthodontics & Dentofacial Orthopedics, Sharavathi Dental College & Hospital, Shivamogga, India; 5 Associate Professor, Department of Orthodontics & Dentofacial Orthopedics, Sharavathi Dental College & Hospital, Shivamogga, India; 6 III Year Post-Graduate Student, Department of Orthodontics and Dentofacial Orthopaedics, Sharavathi Dental College and Hospital, Shivamogga, Karnataka, India

## Abstract

**Background:**

To find out the frequency of extraction in general, in Class I, Class II Class III patients, and to compare the frequency of extraction among sex and age.

**Material and Methods:**

550 cases were selected retrospectively having detailed case history, complete records of facial photographs, lateral cephalogram, orthopantomographs and study models. Frequency of extraction was evaluated separately for class I, class II and class III malocclusion and for sex and ages, using the records collected.

**Results:**

Show that there was 59.80% of extraction in general. Comparison of sex shows that there were 66.60 of extraction in females. The mean age of males for extraction was 17.85 +/- 4.18 and the mean age of females was 18.36 +/_ 4.89. Among all the groups, Class I malocclusion shows 89% of extraction.

**Conclusions:**

There was higher frequency of extraction comprising in general. Comparison of sex shows that there was higher frequency of extraction in females. Comparison of age shows that extraction frequency is more in late adolescent period. Among all the groups, Class I malocclusion shows higher frequency of extraction.

** Key words:**Extraction, frequency, malocclusion.raumatic neuroma; palisaded encapsulated neuroma; oral palisaded encapsulated neuroma.

## Introduction

For more than 100 years, soon after that the practitioners recognized that orthodontic treatment can influence the patients’ profile and esthetics, the extraction of teeth in orthodontics has been a matter of debate (1). In the early 20th century, Edward Angle and his followers believed that extraction destroyed the possibility of ideal occlusion or esthetics (2). As it become clear that arches could and did collapse after expansion despite efforts to produce ideal function, extraction was reintroduced in 1930s in an attempt to overcome relapse problems. By mid-century, extraction had become common place among orthodontists using tweeds modification of edgewise appliance. Tweed has advocated the extraction of 4 premolars to attain facial esthetics and denture similar to those in non orthodontic normal’s (3). To attain this tweed advocated that the mandibular incisor in relation to the basal bone should be 90+/- 5 degrees. The Begg technique was introduced in Australia and many orthodontists who had not used edgewise adopted the Begg approach and began to extract more frequently and the percentage of orthodontic patients with extraction reached a peak (2). Orthodontic treatment by removing teeth had been widely accepted for many types of patients for better long term stability, but non-extraction treatment have again gained widespread popularity with the concern of condylar displacement, narrowed smiles with dark corners, and dished-in profiles with extraction (4,5). Since then extraction percentages have declined noticeably. Extraction frequency is used as a statistical measure describing the number of orthodontic patients having permanent tooth extraction, and it is expressed as a percentage of total treatment samples. It is an unemotional statistic reflecting the sum of all the variables associated with the extraction question. Sometimes, including premolar extraction, produce changes in the facial profile. Therefore, it is useful for the clinical to know the efforts of different treatment options and what they offer to the patients.

-Aims and Objectives

1. To find out the frequency of extraction in S.D.M. College of Dental Sciences and Hospital, Dharwad. for 5 years from 2007 to 2012.

2. To find out the frequency of extraction in Class I, Class II Class III patients.

3. To find out frequency of extraction among age and sex.

## Material and Methods

The records for this investigation were drawn retrospectively over a period of five years from S.D.M. College of Dental Sciences and Hospital Sattur, Dharwad, Karnataka, India from year 2007 to 2012.

The records involved pretreatment study models and pretraced lateral cephalograms which were traced by the respective postgraduate to whom the case was allotted. The treatment plan was decided by the same head of the department for all the five years.

Case selection was based on the following criteria:

1. Patients without any history of orthodontic treatment

2. Age range between 10 – 23 years

3. None of the cases had congenital and dentofacial anomalies or significant facial asymmetries

4. Cases involving surgical treatment were excluded.

-Subject and Methods

Based on inclusion criteria a total of 550 cases were selected having complete records. For all the cases a detailed case history was taken along with facial photographs lateral cephalograms, orthopantamographs, and study models. All cephalograms were obtained on the same cephalometric unit [PMHFCC proline with a cephalostat, manufactured by planmaca OY, Helsinki, FINLAND, with the same magnification of 1:1.09]. The cassette used was Kodak lanex – Omatic, USA.

All cephalograms were hand traced by the respective postgraduate on an acetate mattracing paper with 2H LEAD PENSIL. The following cephalometric analysis:

From the case history files the age sex and malocclusion group, to which the patient belongs to was recorded. All these values were transferred from the files of the each to the extraction table or nonextraction table ( Table 1). The age and sex of the patient were compared among extraction and nonextraction tables and two main variables were further classified as class I class II class III groups, which were further subdivided into extraction and nonextraction subgroups. So a total of 6 tables were obtained. They were the class I extraction, class II non extraction, class II extraction, class II non extraction, class III extraction, class III non extraction subgroups.

**Table 1:**
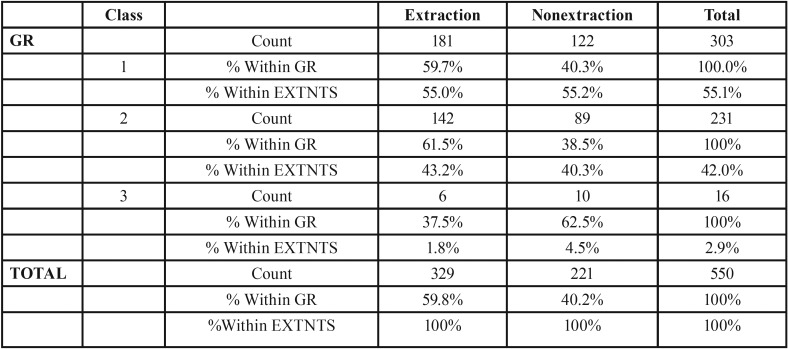
Frequency of extraction in each group.

-Statistical Analysis

Statistical analysis was done using the SSPS software [SPSS for windows XP version 13, SSPS inc, Chicago]. First the independent test was done to compare the subgroups within extraction and non extraction. Then compared among age sex and the 3 malocclusions groups with a multiple comparison bonferroni test to compare among subgroups.

## Results

**Figure 1:**
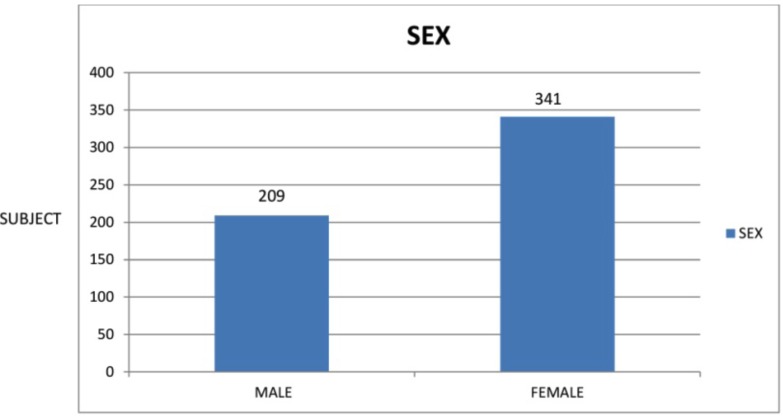
Graph I.

**Figure 2:**
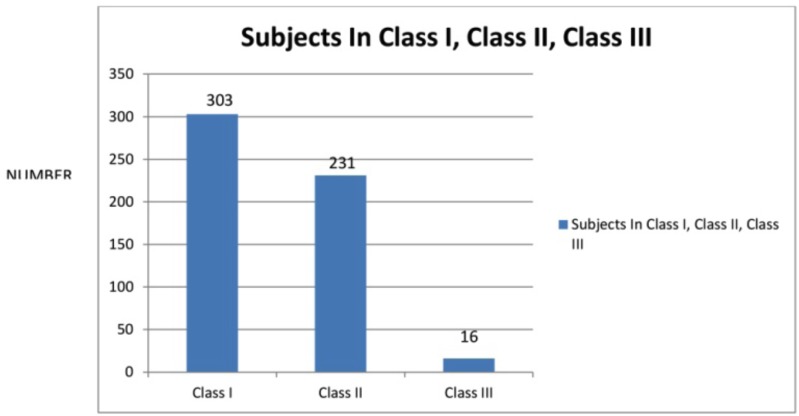
Graph II.

In this study, the total number of subjects was 55 which comprised of 209 [38%] males and 341 females (Fig. 1) they were divided into 3 subgroups according to angles classification as class I class II, class III malocclusion (Fig. 2).

**Figure 3:**
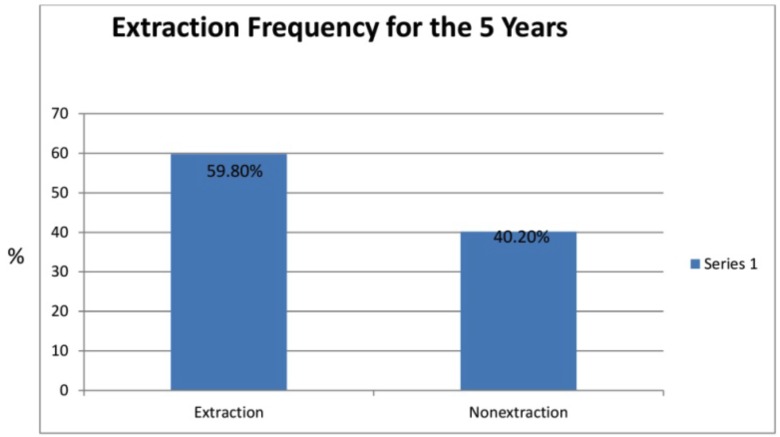
Graph III.

The overall frequency of extraction from the 550 subjects was 329 [59%] and the number of subjects who underwent non extraction was 221 {40.2%] (Fig. 3).

**Figure 4:**
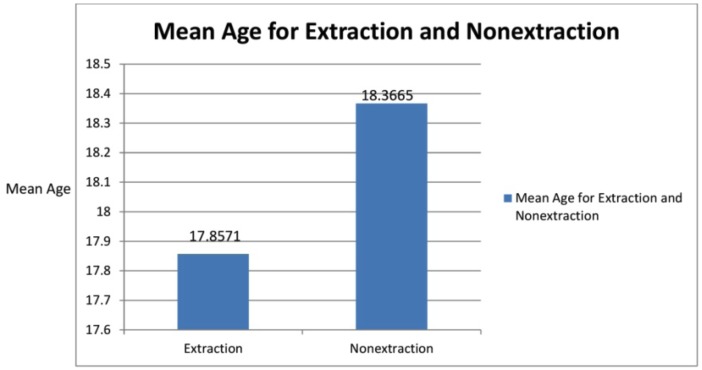
Graph IV.

The mean age of males was 17.85 =/- 4.18 and the mean age of females was 18.56 +/- 4.89 which was not statistically significant [males *p* = .192, females *p* = .206] (Fig. 4).

**Figure 5:**
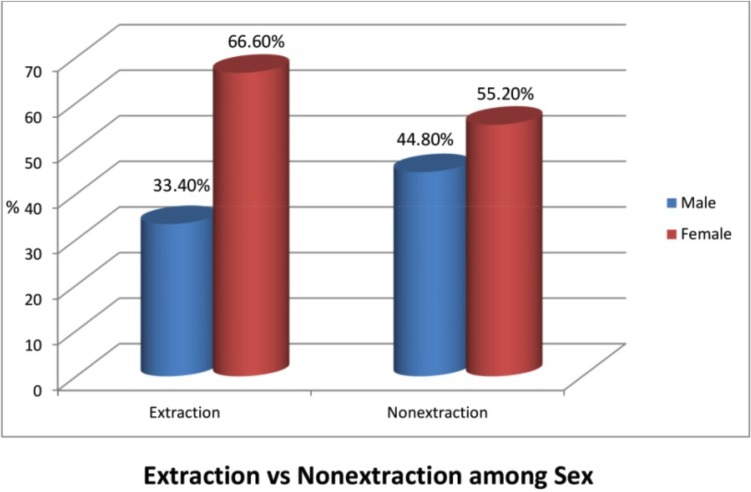
Graph V.

When relating sex with the frequency of extraction for the entire sample, in males the frequency of extraction for the entire sample was 33.4% where as in females the frequency of extraction was 66.6% (Fig. 5). In the non extraction group, the percentage of males was 44.8%, where as in females the percentage of non extraction was 55.5 % (Fig. 5).

**Figure 6:**
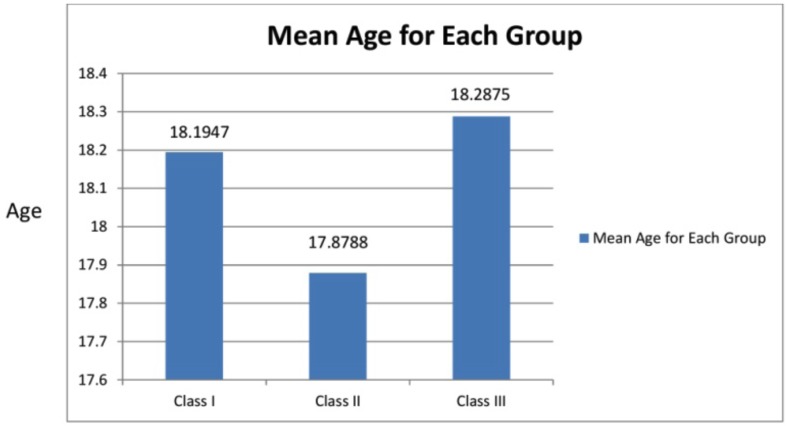
Graph VI.

The mean age of class I, class II, and class III subjects were 18, 19, 17.87 and 18.19 years respectively (Fig. 6).

**Figure 7:**
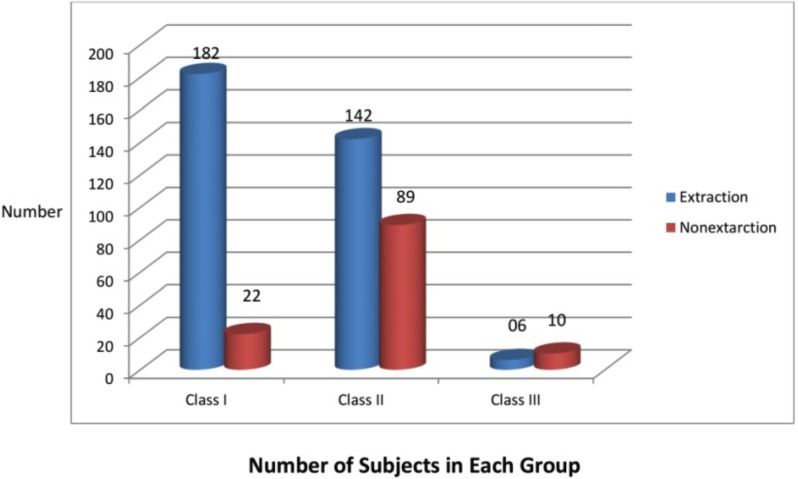
Graph VII.

**Figure 8:**
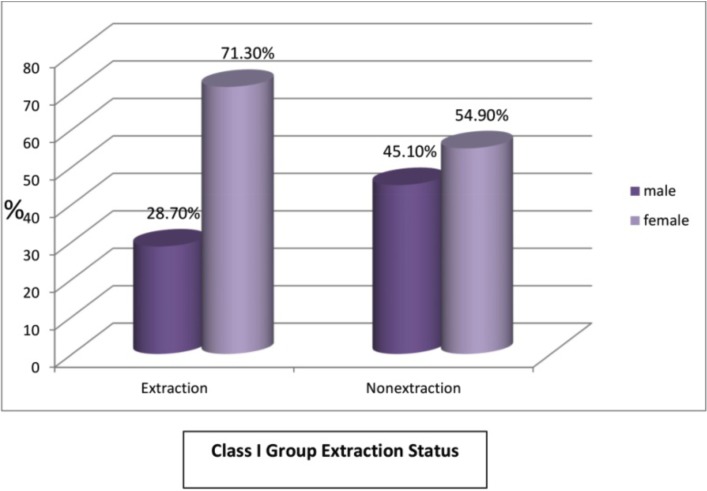
Graph VIII.

Figure 7, the frequency of extraction in each group was from 303 class I subjects 181 subjects (59.73%) underwent extractions and 122(40.26%) underwent the non extraction treatment. Among these 52 (28.7%) males and 129 (71.3%) females underwent extraction and 55 (45.1%) males and 67 (54.9%) females underwent the non extraction protocol ( Table 2) (Fig. 8).

**Table 2:**
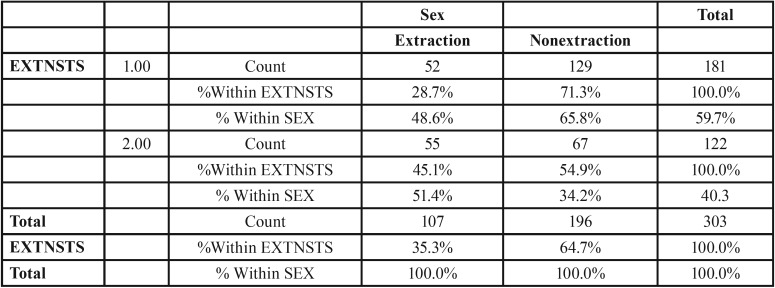
Class I and sex extraction status. extraction status sex cross tabulation.

**Figure 9:**
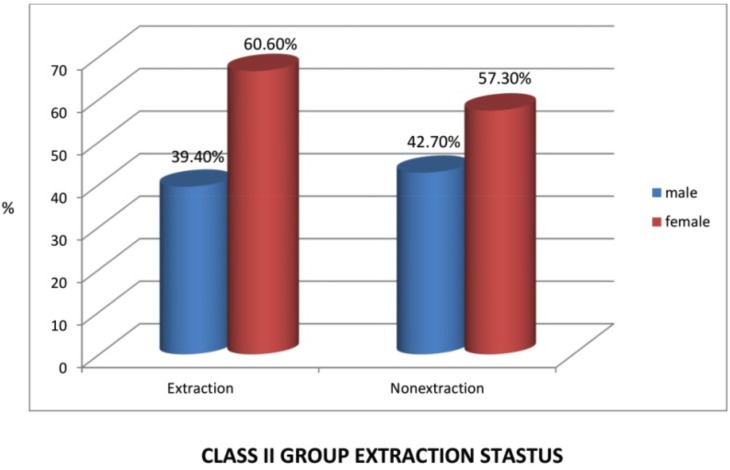
Graph IX.

**Figure 10:**
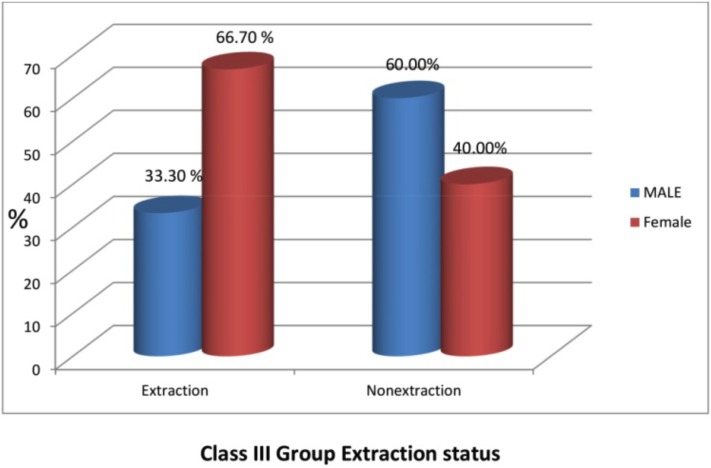
Graph X.

Figure 7, from 231 class II subjects, 142 (61.47%) subjects underwent extraction and 89 (38.52%) subjects underwent the non- extraction treatment (Fig. 9) among these 56 (39.4%) males and 86 (60.6%) females underwent extraction and 38 (42.7%) males and 51 (57.3%) females underwent the non- extraction protocol. ( Table 3) from 16 class III subjects, 6 (37.5%) subjects underwent extraction and 10 (62.5%) subjects underwent non- extraction (Fig. 10) among these 2 (33.3%) males and 4 (66.7%) females underwent extraction and 6 (60%) males and 4 (40%) females underwent nonextraction (Table  Table 4).

**Table 3:**
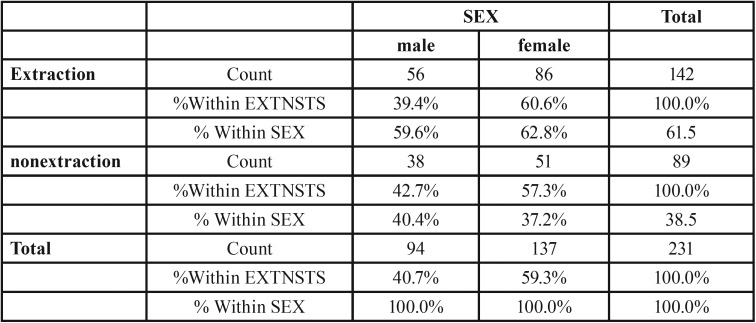
Class II extraction status. extnsts: sex cross tabulation.

**Table 4:**
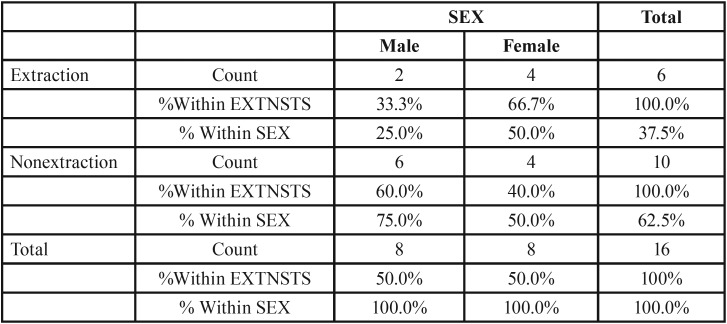
Class III extraction status. extnsts: sex cross tabulation.

## Discussion

Clinicians and researchers are interested in determining the basis of the clinical judgments that are made during the diagnosis and treatment planning of orthodontic cases. Although a number of these judgments are subjective and systematic, hence quantifiable.

In this retrospective study with a sample size of 550, it was found that 329 (59.8%) of the subjects underwent extraction and 221 (40.2%) subjects underwent nonextraction (Fig. 1). This was because majority of the patients came to the orthodontic clinic with complaint of protrusive lips, proclined upper and lower incisors and crowding of either the upper or lower teeth, which could not be corrected solely by nonextraction as it would worsen the profile. Also the patients who had a mildly convex profile prefer a straight profile which was related to their ethnic background as most of the patients were Indians. This was in concordance with a study done by Siddhartha Dhar *et al.* (7).

Among the total of 550 subjects 341 were females (62%) and the remaining 209 were males (38%). This is due to the fact that females were more concerned about their appearance and hence motivated for the orthodontic treatment.

The mean age in extraction group was 17.85 +/- 4.18 years and the mean age for nonextraction was 18.36+/-4.89 years. So age was not a statistically significant factor for the frequency of extraction in the entire sample (Fig. 3). Among the total 329 subjects who had undergone extraction, 66.6% were females and the rest 33.4% were males. This is because females preferred a straight profile while most of the males who had a mildly convex profile were satisfied with their profile (Fig. 4).

Based on Angle’s classification of malocclusion the subjects were divided into Class I, Class II and Class III groups (Fig. 2). Among the Class I subjects (303), 181 subjects underwent extraction and remaining 122 were treated with the non extraction protocol. The most common malocclusion for these patients was crowding and bimaxillary protrusion. Both of these are associated with tooth size arch length discrepancy so these patients were mostly treated by means of extraction of the four first premolars since intentional widening or expansion of the dental arches often is avoided especially when standard edgewise appliance are used, because of the known tendency to relapse according to McNamara.

The present study among the 181 class 1 extraction subgroup (Fig. 8) majority of the females 129 (71.3%) were South Indians with bimaxillary protrusion having a convex profile including few Chinese females. Whereas the Class I non extraction subgroup involved Indian males and females with mildly convex teeth and less crowding of teeth compared to the Class I extraction subgroup.

So it is necessary to take into consideration the patients’ ethnic background, skeletal, dental and physiological age, the function and malformation of teeth and jaws and the soft tissue configuration of the face. Thought the mean age of the Class I extraction group 18.81 years, the tooth size arch length discrepancy -2.33 mm and the proclination of lower incisor to NB of 9.5mm in the Class I extraction group was very highly significant (*p*=0.001) ( Table 2) compared to the non extraction group with tooth size arch length discrepancy of 0.5mm and lower incisor to NB of 6.86mm. Also the mean age of both the Class I extraction and non extraction subgroup was beyond the adolescent growth spurt for mandibular growth to take place.

## Conclusions

1. The frequencies of extraction for 5 years from 2007 to 2016 in the S.D.M. College of Dental Sciences and Hospital of orthodontic department from a sample size of 550 subjects was 59.8% of 329 subjects who underwent extraction and 40.2% with 221 subjects who underwent nonextraction line of treatment comprising higher frequency of extraction in general.

2. The frequency of extraction for class I malocclusion comprising 303 subjects were [59.73%] and 181 subjects who underwent extraction and 122 [40.26%] who underwent nonextraction line of treatment. The frequency of extraction for class II malocclusion comprising 231 subjects were [61.47%] and 142 subjects who underwent extraction and 89 [38.52%] who underwent nonextraction line of treatment. The frequency of extraction for class III malocclusion comprising 16 subjects [37.5%] and 6 subjects who underwent extraction and 10 [62.5%] who underwent nonextraction line of treatment shows that among all the groups, Class I malocclusion shows higher frequency of extraction.

3. The mean age of males was extraction was 17.85 +/- 4.18 and the mean age of females was 18.36 +/_ 4.89 which was not statistically significant [males *p* = .192, females *p* = .206]. (Fig. 4) showing extraction frequency is more in late adolescent period.

4. While relating sex with the frequency of extraction for the entire sample, in males the frequency of extraction was 33.4% where as in females the frequency of extraction was 66.6% (Fig. 5). In the non extraction group, the percentage of males was 44.8%, where as in females the percentage of nonextraction was 55.2% (Fig. 5) shows that there was higher frequency of extraction in females.
